# Extracellular Vesicles as Delivery Vehicles for Non-Coding RNAs: Potential Biomarkers for Chronic Liver Diseases

**DOI:** 10.3390/biom14030277

**Published:** 2024-02-26

**Authors:** Arianna Ferro, Gabriele Saccu, Simone Mattivi, Andrea Gaido, Maria Beatriz Herrera Sanchez, Shafiul Haque, Lorenzo Silengo, Fiorella Altruda, Marilena Durazzo, Sharmila Fagoonee

**Affiliations:** 1 Department of Medical Sciences, University of Turin, 10126 Turin, Italy; arianna.ferro@unito.it (A.F.); gabriele.saccu@unito.it (G.S.); simone.mattivi@unito.it (S.M.); andrea.gaido@unito.it (A.G.); marilena.durazzo@unito.it (M.D.); 2 2i3T, Società per la Gestione Dell’incubatore di Imprese e per il Trasferimento Tecnologico, University of Torino, 10126 Turin, Italy; maria.herrera@2i3t.it; 3 Molecular Biotechnology Centre “Guido Tarone”, 10126 Turin, Italy; lorenzo.silengo@unito.it (L.S.); fiorella.altruda@unito.it (F.A.); 4 Research and Scientific Studies Unit, College of Nursing and Health Sciences, Jazan University, Jazan 45142, Saudi Arabia; shafiul.haque@hotmail.com; 5 Centre of Medical and Bio-Allied Health Sciences Research, Ajman University, Ajman 13306, United Arab Emirates; 6 Gilbert and Rose-Marie Chagoury School of Medicine, Lebanese American University, Beirut 1102 2801, Lebanon; 7 Institute for Biostructure and Bioimaging, National Research Council (CNR), Molecular Biotechnology Centre “Guido Tarone”, 10126 Turin, Italy

**Keywords:** chronic liver diseases, non-coding RNAs, diagnosis, extracellular vesicles, biomarkers

## Abstract

In recent years, EVs have emerged as promising vehicles for coding and non-coding RNAs (ncRNAs), which have demonstrated remarkable potential as biomarkers for various diseases, including chronic liver diseases (CLDs). EVs are small, membrane-bound particles released by cells, carrying an arsenal of ncRNAs, including microRNAs (miRNAs), long non-coding RNAs (lncRNAs), and other ncRNA species, such as piRNAs, circRNAs, and tsRNAs. These ncRNAs act as key regulators of gene expression, splicing, and translation, providing a comprehensive molecular snapshot of the cells of origin. The non-invasive nature of EV sampling, typically via blood or serum collection, makes them highly attractive candidates for clinical biomarker applications. Moreover, EV-encapsulated ncRNAs offer unique advantages over traditional cell-free ncRNAs due to their enhanced stability within the EVs, hence allowing for their detection in circulation for extended periods and enabling more sensitive and reliable biomarker measurements. Numerous studies have investigated the potential of EV-enclosed ncRNAs as biomarkers for CLD. MiRNAs, in particular, have gained significant attention due to their ability to rapidly respond to changes in cellular stress and inflammation, hallmarks of CLD pathogenesis. Elevated levels of specific miRNAs have been consistently associated with various CLD subtypes, including metabolic dysfunction-associated steatotic liver disease (MASLD), metabolic dysfunction-associated steatohepatitis (MASH), and chronic hepatitis B and C. LncRNAs have also emerged as promising biomarkers for CLD. These transcripts are involved in a wide range of cellular processes, including liver regeneration, fibrosis, and cancer progression. Studies have shown that lncRNA expression profiles can distinguish between different CLD subtypes, providing valuable insights into disease progression and therapeutic response. Promising EV-enclosed ncRNA biomarkers for CLD included miR-122 (elevated levels of miR-122 are associated with MASLD progression and liver fibrosis), miR-21 (increased expression of miR-21 is linked to liver inflammation and fibrosis in CLD patients), miR-192 (elevated levels of miR-192 are associated with more advanced stages of CLD, including cirrhosis and HCC), LncRNA HOTAIR (increased HOTAIR expression is associated with MASLD progression and MASH development), and LncRNA H19 (dysregulation of H19 expression is linked to liver fibrosis and HCC progression). In the present review, we focus on the EV-enclosed ncRNAs as promising tools for the diagnosis and monitoring of CLD of various etiologies.

## 1. Introduction

Chronic liver diseases (CLDs) comprise a wide range of conditions characterized by the progressive deterioration of hepatic functions. The initial damage can be due to infections, heavy alcohol consumption, or dysmetabolic or autoimmune mechanisms. Regardless of its origin, damage leads to a continuous process of inflammation, destruction, and regeneration of the liver parenchyma, inducing the formation of fibrotic tissue. Sustained progression of fibrosis may even lead to cirrhosis and hepatocellular carcinoma (HCC) [1].

CLDs affect around 1.5 billion people worldwide. They primarily include non-alcoholic fatty liver disease (NAFLD) and non-alcoholic steatohepatitis (NASH), recently renamed metabolic dysfunction-associated steatotic liver disease (MASLD) and metabolic-dysfunction-associated steatohepatitis (MASH), respectively, and consequently employed herein, followed by viral hepatitis, alcoholic liver disease (ALD), autoimmune hepatitis (AIH) and cholestatic liver diseases [2]. Because of its high prevalence, CLD is one of the main causes of mortality and morbidity across the world, especially in developing countries [3]. In particular, MASLD is the most widespread CLD. It affects 30% of the population, but this rate is expected to increase further [4]. MASLD-correlated mortality ranges between 0.5% and 9%, with liver complications (e.g., cirrhosis, hepatocellular carcinoma (HCC)) and the augmented risk of cardiovascular diseases greatly contributing to this rate [5]. On the other hand, ALD prevalence is 4.8% worldwide, and it is more common among males compared to females (2.9% vs. 0.5%, respectively). Alcoholic liver cirrhosis occurs in 32.9% of cases and frequently causes death (23.9%) [6]. Chronic viral hepatitis, caused by viruses such as hepatitis B virus (HBV) or hepatitis C virus (HCV), induces a chronic inflammatory state of the liver. Although there are many treatment options for HBV and HCV infections, these cause 1.1 million deaths and 3 million new infections every year combined (European Centre for Disease Prevention and Control, https://www.ecdc.europa.eu/en/news-events/world-hepatitis-day-2023-need-scale-efforts-address-hepatitis-b-and-c; last visited on 13 February 2024). Other chronic liver diseases are quite rare, and their prevalence ranges from 4.8 to 42.9 per 100,000 persons for AIH, from 0 to 31.7 per 100,000 for primary sclerosing cholangitis (PSC), and from 1.91 to 40.2 per 100,000 for primary biliary cholangitis (PBC) [7].

At the end stage of the disease, complications of cirrhosis such as ascites, gastrointestinal bleeding, and encephalopathy may occur, impairing the patients’ quality of life and progressively leading to liver failure and/or HCC. In these cases, liver transplantation (LT) remains the only therapeutic option [8]. Therefore, early diagnosis and prompt intervention are challenging to improve the prognosis of the disease.

Liver biopsy is currently considered the gold standard for the assessment of liver fibrosis and, hence, for the diagnosis of CLD. However, this procedure is quite invasive and expensive and can cause serious complications, such as bleeding and sampling errors [9]. Thus, during recent years, the interest in developing alternative less invasive, simple, and cost-effective diagnostic tests, including radiological procedures, biochemical scoring, and serum biomarkers, has risen considerably [10]. In the present review, we will provide an update on the biomarkers available or under development for the diagnosis and prognosis of CLD, both at clinical and preclinical levels, with special emphasis on non-coding RNA (ncRNA) biomarkers enclosed within circulating extracellular vesicles (EVs).

## 2. Biomarkers

The original definition of biological markers (biomarkers) was that of “cellular, biochemical or molecular alterations which are measurable in human tissues, cells or fluids” [11]. More recently, the Food and Drug Administration (FDA) and National Institutes of Health (NIH) have revised this definition to a “defined characteristic that is measured as an indicator of normal biological processes, pathogenic processes or responses to an exposure or intervention” [12].

At the clinical level, biomarker research involves tools and technologies, which can help in understanding the various phases of a disease, from prediction to diagnosis and prognosis. Biomarkers can be further classified into diagnostic biomarkers, monitoring biomarkers, predictive biomarkers, and prognostic biomarkers [13]. Diagnostic biomarkers are used to detect or confirm the presence of a disease. Their clinical performance is evaluated through comparison with other diagnostic tests and the calculation of clinical sensitivity and specificity. A perfect diagnostic biomarker test should aim at identifying all patients with the disease (100% sensitivity) and excluding all patients without the illness (100% specificity) [12,14,15]. On the contrary, monitoring biomarkers are used for assessing the status of the disease. For this reason, they are repeatedly measured over time in order to assess disease progression and the eventual response to a treatment, either favorable or unfavorable [12,13]. Predictive biomarkers are instead used to identify individuals who are more likely to experience a particular effect (either positive or negative) compared to other similar individuals. They are widely applied in interventional trials, in taking patient care decisions, and to study the effect of exposure to environmental factors on the population. [12]. Lastly, prognostic biomarkers are used to identify the likelihood of a future clinical event (including illness progression, relapses, and death) in patients affected by a certain disease [12,14]. Some authors also consider response and safety biomarkers: the former change in response to a pharmacological or an environmental agent exposure, while the latter are used to identify the possible toxicity of that agent [12].

**Figure 1:**
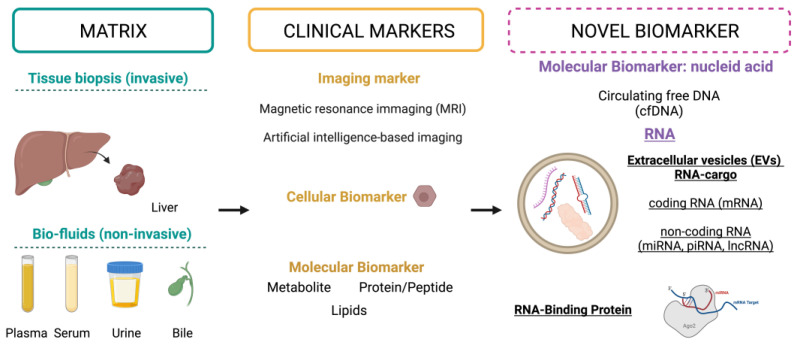
A comprehensive schematic representation highlighting the diverse sample types from which biomarkers can be extracted to assess the status of CLD. The clinical approaches employed and the novel markers that have been investigated are illustrated. Created with BioRender.com (https://app.biorender.com/; accessed on 20 December 2023), license number: Y267V28IT.

According to their intrinsic characteristics, biomarkers can be further distinguished as imaging biomarkers, cellular biomarkers, and molecular biomarkers (Figure 1) [16]. An imaging biomarker is a characteristic that allows us to objectively measure a biologic feature or response to therapy through an image [16]. They are widely used in clinical settings because of their low invasiveness and high cost-effectiveness. Examples include the measurement of proton density fat fraction with Magnetic Resonance Imaging (MRI) for the detection of liver fat and to diagnose MASLD or the use of artificial intelligence-based imaging biomarkers for the prognosis of the pejoration of chronic liver diseases into cirrhosis [17,18]. On the other hand, cellular biomarkers are measurable indicators that allow cells of interest to be isolated and characterized on the basis of their morphology and physiological states, such as through the expression of antigens, to enable their isolation using specific methods like, for instance, fluorescent-activated cell sorting [16]. The surface marker CD133 is an example and enables the identification of liver cancer stem cells [19]. In recent years, however, clinical and research attention has growingly focused on molecular biomarkers. Molecular biomarkers can be identified in biological samples such as serum, plasma, and tissues and comprise a wide range of molecules, different in size and origin, which can be classified based on their chemical nature or their omics profile, including genomics, transcriptomics, proteomics, and metabolomics, and which can be extracted with big data analysis using machine learning and deep learning technologies [16,20,21].

Regardless of the category to which they belong, biomarkers show numerous advantages in clinical settings. Firstly, they are by definition objective. This means that they do not necessarily correlate with patients’ feelings and clinical status, but they impartially represent a condition. The concept of objectivity brings with it two other essential characteristics of a biomarker: measurability and repeatability. Biomarkers should be measurable using international standard methods and show repeatability and reproducibility [22]. Importantly, biomarkers must be validated. A biomarker validation process is quite complex and includes two types of evaluations: analytical and clinical. The former is an internal validation and it allows us to assess biomarker performance through different metrics (e.g., sensitivity, specificity, accuracy, precision), while the latter is an external validation which evaluates the usefulness of the biomarker in the clinical setting [23]. Thus, a validated biomarker implies not only reliability but also clinical relevance.

Despite their potential as valuable diagnostic and prognostic tools, the use of biomarkers is not exempt of challenges. Laboratory errors, confounding factors, patients’ features, and high costs may significantly impact their effectiveness [24]. To overcome these obstacles, rigorous training for laboratory personnel and standardized protocols are essential [22].

### Current Research and Approaches to Finding New Biomarkers

In the last decades, the colossal benefits of biomarker utilization in clinical practice have incited the search for new potential disease-related molecules. Meanwhile, the spread of novel high-throughput technologies, such as the “omics” techniques, has led to the detection of novel biological entities and to the analysis of big biological data in an efficient way. During recent years, both cell-free and EV-enclosed molecular biomarkers have received consistent attention. In the present review, we will focus mainly on the biomarker utility of biomolecules enclosed in EVs.

Currently, the assessment of CLD markers relies on patient-related parameters, including age, body mass index, comorbidities, and serum markers such as AST and ALT [25]. Additional research has investigated biomarkers that may represent the transition from CLD to fibrosis, including platelet count, hyaluronic acid, type IV collagen, bilirubin, and tissue inhibitor of matrix metalloproteinase 1 (TIMP1) [25]. Recently, circulating cell-free DNA (cfDNA) has emerged as a novel non-invasive biomarker for CLD. In particular, the presence of cfDNA in the bloodstream may represent a promising marker of end-stage CLD with HCC [26].

Interestingly, extracellular RNA classes offer a potential source of novel biomarkers for various pathophysiological states, particularly in CLD (Figure 1). Specifically, protein-coding RNA (mRNA) captures the gene signature of a particular cell across both physiological and pathological conditions. Beyond their fundamental role in carrying genetic information, RNA molecules undergo intricate regulatory processes at both the transcriptional and post-transcriptional levels by non-coding RNA (ncRNA) [27]. Based on its length, ncRNA can be categorized into different subclasses, including small ncRNAs (<200 bp), represented by microRNAs (miRNAs), PIWI-interaction RNA (piRNA), siRNA, and tRNA, and long ncRNAs (lncRNAs, >200 bp), such as circular RNAs (circRNAs), small nuclear RNAs (scRNAs), and transfer RNA-derived small RNA (tsRNA) [28]. The release of these RNA molecules throughout the disease progression via diverse mechanisms in biological fluids may serve as a hallmark of disease severity. RNA can be actively released into EVs or found extracellularly, bound to protein or lipid complexes, such as proteolipid, lipoprotein, or other RNA-binding proteins [29]. The piRNAs, a novel type of small ncRNA that interact with PIWI subclass Argonaute proteins, and circRNA have also recently emerged as a potential biomarker for several pathological conditions [29].

Therefore, the diverse mechanisms of RNA release contribute to the prolonged stability of circulating RNAs, thus enabling the detection of distinct disease stages and establishing a promising molecular diagnostic approach. Circulating RNAs, especially miRNAs, show unusual stability due to their packaging in EVs, discussed in this review, or their secondary structure and size or their binding to ribonucleoprotein complexes [30].

## 3. Extracellular Vesicles and Nanoparticles

### 3.1. Biogenesis and Characterization

**Figure 2:**
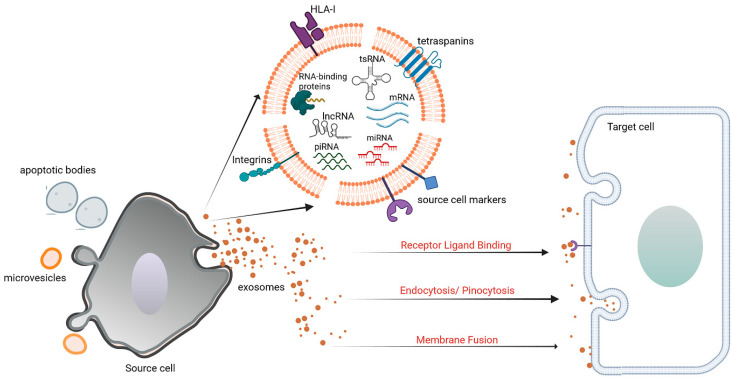
Extracellular vesicle release and clearance. Depending on their biogenesis, EVs (including exosomes, microvesicles, and apoptotic bodies) released by the source cell carry molecular information to the recipient cell. Apart from proteins, lipids, and metabolites, different RNA species are present in EVs. Some of the modalities of EV internalization in the recipient cell are shown. Image created with BioRender.com (https://app.biorender.com/; accessed on 20 December 2023), license number: IE267V3T7X.

EVs are heterogeneous, small nano-sized, lipid bilayer membrane-enclosed particles which shuttle biologically active substances, including nucleic acids, proteins, lipids, and metabolites, and are released by all cell types of the body [31]. The biogenesis of EVs is primarily driven by the endosomal sorting complex required for transport (ESCRT) machinery, with Alix playing a crucial role in its regulation [32]. These particles, coined as “Extracellular Vesicles” in 2011, can be further distinguished into three categories depending on their size and biogenesis: exosomes, microvesicles, or apoptotic bodies (Figure 2) [33]. EVs play an important function in long-distance intercellular communications, both under physiological and pathological conditions. Their low immunogenicity and cytotoxicity render EVs as attractive platforms for designing new drug delivery vectors. The therapeutic potential of EVs is under deep scrutiny, especially as potential tools for personalized medicine for several diseases. For instance, regarding liver diseases, EVs derived from different sources (serum, stem cells, hepatocytes) were tested in cellular or preclinical models of human liver diseases, such as MASLD, for their therapeutical effects, as extensively described recently [34,35,36]. In particular, what is evident from the studies performed is that EVs derived from stem cells are capable of counteracting inflammation and fibrosis by dampening hepatic stellate cell activation and the expression of profibrogenic genes. In a carbon tetrachloride-induced rodent model of liver fibrosis, e.g., EVs derived from mesenchymal/stromal stem cells (MSCs) from several sources were employed. Native EVs derived from human umbilical cord MSCs decreased liver injury by dampening the transforming growth factor (TGF)-β1/Smad signaling pathway and inhibiting the epithelial-to-mesenchymal transition [37]. EVs modified to over-express miRNAs such as miR-181-5p or miR-122 conferred enhanced modulation of liver inflammation and fibrogenesis in the preclinical models [38]. In the setting of non-alcoholic steatohepatitis (NASH), rats fed a high-fat diet for four weeks and treated with amniotic MSC-derived EVs had reduced activation of pro-inflammatory M1 macrophages and expression of pro-inflammatory cytokines including TNF-alpha, IL-1-beta, and IL-6 [39]. Moreover, using EVs derived from human liver stem cells, which have been extensively characterized in our lab both in vitro and in vivo regarding liver diseases, Bruno et al. showed how the treatment of NASH mice (induced with methionine- and choline-deprived diet) with these nanoparticles harboring anti-inflammatory and anti-fibrogenetic molecules could improve the liver phenotype compared to controls [40,41,42,43].

Due to their biocompatibility, EVs have also been engineered to specifically deliver therapeutic cargo to cancer cells. For instance, Kim et al. recently described a rapamycin-induced protein–protein interaction cargo delivery system to modify EVs by using the binding affinity between the FKBP12–rapamycin-binding protein (FRB) domain and FK506-binding protein (FKBP) [44]. These EVs were functionally delivered to recipient refractory cancer cells, indicating that this system is promising for the delivery of therapeutic cargo specifically to target cells.

Thanks to their cargo, which provides a real-time molecular snapshot of the source cell, EVs are also useful in the search for biomarkers of diseases which induce changes in the amount as well as composition of EVs with respect to a healthy condition [45,46]. EVs are dependent on the types and functional states of the source cells, and it is not always easy to determine the biogenetic origin of the EVs due to difficulty in finding cell-type-specific markers on the EVs’ surface. To further complicate this issue, apart from EVs, cells also release extracellular particles which lack a lipid bilayer membrane, termed non-vesicular extracellular nanoparticles (NVEPs). The identification of these particles has been possible due to advances in technology and detection methods. Importantly, the NVEPs include lipoprotein particles, nucleosomes, and vaults, as well as the recently identified exomeres and supermeres [47]. These NVEPs, of yet unknown biogenesis, contain RNA and DNA molecules as well as proteins, mainly involved in metabolism [48]. The supermeres, for instance, are replete with molecules that may serve as disease biomarkers. All these nanoparticles are useful assets in finding biomarkers which would be able to detect the surge in chronic liver diseases very early.

### 3.2. Liver in EV Release and Clearance

The liver is a vital organ relying on the finely tuned and coordinated interaction among its highly heterogeneous cell types to respond to insults and stress to which it is exposed constantly. The hepatocytes constitute the bulk of the liver lobes and, together with the cholangiocytes, represent the parenchymal cell fraction of the liver. The non-parenchymal fraction consists of hepatic stellate cells, Kupffer cells, or liver sinusoidal endothelial cells (LSECs). All the liver cell types and the various immune cell populations present in this organ can release large quantities of EVs under stress [49]. Liver-derived EVs contain specific cargo, including certain molecules that are characteristic of donor cells (Table 1). EVs secreted into the extracellular space may be captured by neighboring cells or carried away by the blood to distant tissues, where they exert paracrine, endocrine, and, sometimes, autocrine effects.

The recipient cell can utilize different uptake mechanisms, including endocytosis (clathrin- and caveolin-dependent), macropinocytosis, phagocytosis, and lipid raft-mediated uptake, with the former being the most prevalent [50]. The liver is the main site of EV clearance, and EVs were seen to accumulate in the liver of rodents as early as 10 min following intravenous injection in rodents and can remain for hours or days (Table S1). Because hepatocytes exhibit polarity, the uptake and release of EVs, as well as their number and composition, may vary between the apical side (facing the bile, referred to as apical EVs) and the basolateral side (facing the blood, referred to as basolateral EVs) [69]. Recent data point out the role played by the Vesicular-Associated Membrane Proteins (VAMPs), in particular by the VAMP8/Endobrevin, in the basolateral release of EVs [69]. Other mechanisms may be involved, and further studies are needed to decode the different sorting strategies employed by the basolateral and apical sides of the polarized epithelial cell.

The EV scavenger function is carried out mainly by Kupffer cells followed by LSECs and hepatocytes [70]. Hints from preclinical studies, using SPECT/CT imaging, indicated that medium and small ^99m^Tc-HYNIC-Duramycin-labelled EVs could be preferentially taken up by the liver macrophages and LSECs, respectively.

Several fates of internalized EVs have been hypothesized. EVs may escape the endosomal compartment for the functional delivery of biomolecules to other organs; the EV cargo can also undergo degradation or repurposing, or the intact EV may be released back into the extracellular space. O’Brien et al. showed how the EV cargo has different destinies inside the cytosol: cytochrome c oxidase subunit 8 (CoxVIII) protein contained in EVs could switch location from the cytosol to mitochondria, and EV histone H2B RNA can be translated [71]. Importantly, the underlying liver disease had a profound impact on the dynamics of EV release and uptake by the liver. In this regard, we have observed a significant increase in the number of circulating EVs in mice undergoing bile duct ligation, with respect to controls [53]. This was also observed in other liver pathologies in humans or in rodents, thus suggesting that apart from EV content, the number of circulating EVs can also be indicative of damage [72,73].

### 3.3. Isolation of EVs and Characterization of Their Molecular Contents

The isolation methods for EVs have undergone significant revolution since the time ultracentrifugation was first employed to prepare EVs, extensively described in [74]. Centrifugation-based methods for EV purification include ultracentrifugation, simple differential ultracentrifugation, and density gradient centrifugation. Size-exclusion chromatography and charge-, antibody-, or magnet-based isolation have gained much interest in the field of EV research. Moreover, microfluidic technologies have offered an integrated platform for a myriad of applications, including EV capture and characterization at the physical and biochemical levels [75]. These micro-devices are equipped for both physical and molecular capturing approaches. Surface-enhanced Raman scattering has recently been incorporated into complex microfluidic devices because of its exceptional sensitivity, stability, rapid readout, and the ability to multiplex [76]. The isolation technique employed has a significant influence on the outcome of the downstream analyses of EV cargo. EV quantification is performed using several instruments available, ranging from the Nanosight tracking analysis tool to flow cytometry and Raman spectrometry [74].

The preclinical data on EVs have boosted the use of these therapeutic vehicles in clinical-grade format for use in several clinical trials. However, the need for huge quantities of EVs to obtain therapeutic benefits, whilst preserving their physico-chemical properties, has solicited some serious reconsideration regarding the purification technique to be employed in the clinical setting. Using a 3D reactor may be a valid solution for the large-scale production of therapeutic-grade EVs isolated from MSCs stimulated under different conditions, for instance [77,78].

In the quest for diagnostic and prognostic biomarkers for human diseases, which require timely intervention in order to hinder the dysfunctionality of vital organs or to follow the outcome of therapeutic regimens, the bioactive contents of EVs hold a very important position. EVs, due to their accessibility from biological fluids (plasma, serum, urine, saliva, breast milk, bronchoalveolar lavage fluid, pleural effusion, cerebrospinal fluid, and amniotic fluid) and their molecular landscape mirroring disease states, fulfil the criteria of non-invasive biomarkers, thus soliciting research in the area of precision medicine [79,80].

EVs are enriched in nucleic acids, with the RNA cargo skewing towards small RNA transcripts, that is, miRNAs, siRNAs, and piRNAs. Other longer transcripts have been revealed in EVs, including mRNA, lncRNAs, snRNAs, circRNAs, retrotransposons, and pseudogenes [81]. Proteins, lipids, and metabolites also form part of the EV cargo. The most frequently employed methods for the characterization of each of these bioactive molecules are described in Table 2.

EV-enclosed mRNAs, for instance, show several advantages as biomarkers of diseases. These mRNAs are released inside EVs in a particular context (disease state, time) and thus reflect the molecular status of the donor cells. For instance, EV-enclosed mRNAs can be used as biomarkers for cancer detection and drug resistance monitoring.

Characterizing EV RNA profiles poses a significant challenge due to methodological hurdles in the isolation and purification of EVs. These challenges are exacerbated by complex RNA extraction protocols for already-limited RNA quantities within EVs, as well as the intricate nature of the biocomputational analysis required for the RNA data. To add to the complexity of the system, cell-free RNA is bound to non-vesicular particles such as lipoproteins, RNA-binding proteins, or exomeres and can be co-isolated with RNA enclosed inside EVs, hence masking details regarding the origin of the EVs. Despite the hitherto unresolved issues, the EV-based biomarker search has gained much ground and is faring well in all aspects of disease detection and monitoring, both at the preclinical and clinical levels. In this regard, the advancement in “-omics” technologies, particularly massively parallel nucleic acid sequencing, has empowered researchers to engage in a wide spectrum of EV studies, both discovery-based and hypothesis-driven [80]. The use of EV-enclosed RNAs as biomarkers in CLD is discussed below.

## 4. EV-Enclosed Non-Coding RNAs as Biomarkers for Detection and Monitoring of Chronic Liver Diseases

As mentioned above, cell-free endogenous RNA molecules can be detected in human plasma, and they show remarkable stability despite the presence of circulating RNAses [30]. Among the RNA species potentially useful as biomarkers, miRNAs, and to some extent mRNAs despite their being less stable than miRNAs, have gained increasing attention lately due to the extensive use of high-throughput RNA sequencing on patient-derived samples.

The first report demonstrating a diagnostic capacity of circulating miRNAs in hepatic diseases dates back to 2009, when Wang et al. showed that, in a mouse model with induced liver injury, serum levels of miR-122 and miR-192 exhibited a dose-dependent increase that anticipated and correlated with the rise in ALT activity [82]. Since then, numerous studies have been carried out in order to understand the possible role of miRNAs in CLD with various etiologies. For instance, the most abundant hepatic miRNA species, miR-122, appears to be the first altered miRNA detectable during the early development of NAFLD [83]. Human studies suggest that its levels gradually decrease with disease progression towards NASH and fibrosis. MiR-122 was found to be increased in simple steatosis versus healthy liver, whereas it was found to be reduced in NASH compared to simple steatosis [84]. Moreover, Xu et al. demonstrated that the hepatocyte-specific expression of miR-34a aggravated diet-induced NAFL in C57BL/6 mice [85]. Remarkably, they also found that the pharmacological inhibition of miR-34a reversed diet-induced steatohepatitis. This improvement was obtained via the regulation of lipid absorption, lipogenesis, inflammation, apoptosis, and inhibition of fatty acid oxidation sustained by miR-34a. Interestingly, it was also found that the activation of the miR-34a/SIRT1:AMPK pathway leads to mitochondrial dynamics dysfunction in the skeletal muscle in human and experimental NAFLD, thus representing a promising pharmacological target for the treatment of metabolic syndrome [86]. Furthermore, circulating miR-34a levels have been shown to be increased in serum from patients with NASH compared with NAFLD and are positively correlated with histopathological features of the liver, a key feature for a quantitative marker [87,88]. However, circulating cell-free RNA as a potential biomarker can be affected by the following: (1) the increased presence of RNAs derived from red blood cells due to low-level hemolysis occurring during blood sample collection or during their transport and conservation; (2) RNAs released by other blood cells present in the plasma; and (3) a delay in sample processing that may induce RNA degradation.

**Figure 3:**
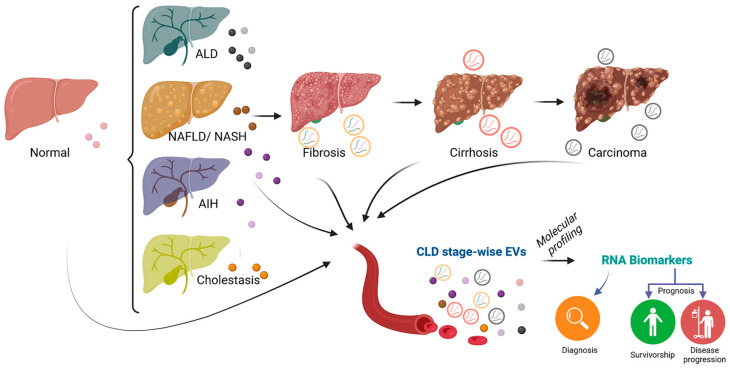
EV-RNA biomarker search for chronic liver diseases. Some of the most common etiologies of CLD are shown. Chronic liver injury leads to the development of fibrosis, which can progress into cirrhosis and eventually into carcinoma at advanced stages. RNA molecules encapsulated within circulating EVs and characterized with high-throughput molecular profiling approaches are important sources of biomarkers for diagnosis as well as for predicting survivorship and disease progression. Image created with BioRender.com (https://app.biorender.com/; accessed on 20 December 2023), license number: TS267V2H8F.

RNA enclosed within EVs has thus become the focus of the biomarker search for its “message in a bottle”-type active release into the circulation under pathological conditions [89]. The RNA loading mechanism into EVs is still under investigation. Some RNA species are exclusively secreted into EVs, and the EV transcriptome does not always reflect the cellular transcriptome [81]. It could present a way to post-transcriptionally regulate intracellular RNA homeostasis [90]. Importantly, mRNA molecules enclosed within the phospholipid bilayer structure of EVs are far more resistant to RNAse activity with respect to their cell-free, protein-bound counterparts, thus opening up the possibility of combining different kinds of RNA species for an improved and more precise biomarker panel [91]. The main EV-enclosed RNAs involved in both preclinical and clinical trials are discussed below in the context of chronic liver diseases of different etiologies, in particular NAFLD/MASLD and NASH/MASH, alcoholic liver diseases (ALDs), liver fibrosis, autoimmune hepatitis (AIH), chronic viral hepatitis, and cholestatic liver diseases (Figure 3). Both lncRNAs and circRNAs are involved in gene expression regulation by the EV *lncRNA-miRNA-mRNA* and EV cirRNA-miRNA-mRNA axes [20]. The sponge-like action of lncRNAs on miRNAs helps to sequester the latter from their target mRNAs. For instance, the lncRNA ASMTL-AS1 can sequester miR-342-3p from its target mRNA, c-Myc, a potent oncogene, thereby promoting the malignancy of HCC after insufficient radiofrequency ablation [92]. On the other hand, EV circRNAs play an important role in tumorigenesis, including tumor immunity, and can be used as biomarkers for predicting cancer patients’ outcomes or for evaluating therapeutic efficacy [93]. In preclinical models, circRNA-100338, for instance, was found to participate in the dissemination of HCC cells and promote invasiveness and angiogenesis, hence metastasis [94]. Understanding the role of EV lncRNAs and EV circRNAs and the axes in which they are involved in CLD is challenging but essential.

### 4.1. NAFLD/MASLD

NAFLD, now known as MASLD, is the most common hepatic illness in Western countries [3]. Its estimated prevalence is 25% in the general population, but it can reach 50–60% in specific populations, such as in obese and diabetic subjects [95]. Actually, MASLD does not represent a single pathology, but it includes several pathological conditions, ranging from simple steatosis to hepatic inflammation (non-alcoholic steatohepatitis, NASH, now named MASH) to liver necroinflammation and fibrosis, which can even evolve into cirrhosis and HCC [3,96].

Presently, there is no accurate method for diagnosing or staging MASLD other than through invasive liver biopsy. Due to the spread of MASLD and its associated morbidity and mortality, research in this field has increasingly focused on finding biomarkers for this disease, especially regarding the use of EVs as a non-invasive tool for its early diagnosis. In this regard, Newman et al. demonstrated the enhanced performance of liver-specific EV miRNAs in assessing MASLD severity [57]. Liver-specific EVs were isolated with anti-asialoglycoprotein receptor 1-mediated immunoprecipitation from MASH, MASLD, and control patients, and their miRNA content was compared to that of global EV-RNA or cell-free RNA. Interestingly, the sorted liver-derived EVs showed enrichment of miR-122, miR-192, and miR-128-3p which was significantly associated with disease severity with respect to the other groups. This study pinpoints the importance of sorting tissue-specific EVs to gather more information on the pathology and to potentially apply the EV RNA contents as stage-wise diagnostic and prognostic biomarkers in the clinical setting, instead of the classical dichotomous grouping of biomarkers into diseased versus healthy conditions.

Several studies have also highlighted an increased amount of circulating EVs in preclinical models. For instance, cholesterol-treated hepatocytes were found to release large quantities of EVs, and this was confirmed in mice fed with high-fat diets [97,98]. This was probably associated with lipid accumulation, which not only stimulated inflammation and fibrogenesis but also enhanced EV secretion [99,100]. Further analysis of EV content in the MASLD setting showed differences in RNA pattern, too. For example, in high-fat-diet-fed mice, a significant decrease in the levels of miR-135a-3p, miR-129b-5p, and miR-504-3p and an increase in those of miR-122-5p in serum EVs were observed [97]. The expression pattern of miR-135a-3p was mirrored in MASLD patients, hence allowing the accurate diagnosis of this condition. Thus, a combination of EV-enclosed miRNAs could develop into a biomarker panel for detecting MASLD and its progression into MASH.

A recent systematic review by Zeng et al. analyzed lncRNA and circRNA patterns in MASLD. Differences were observed in the expression of twenty-two lncRNAs: fifteen of these were upregulated (e.g., NEAT1, lncARSR), while the other six were downregulated (e.g., lncSPARCL1) in patients with MASLD [101]. Similarly, in the study by Sun et al., substantial differences in lncRNA expression profiles between MASLD and normal control tissues were identified [102]. They observed that 535 lncRNAs were upregulated in MASLD samples, while 1200 were downregulated. This finding highlights the intricate regulatory role of lncRNAs in MASLD pathogenesis. Further analysis demonstrated a close concordance between microarray data and PCR quantification, suggesting that these lncRNAs could serve as promising biomarkers for MASLD diagnosis. Among these, NEAT1, MEG3, and MALAT1 showed great potential as biomarkers for the disease. Differentially expressed circRNAs were also observed in patients with MASLD and MASH (e.g., circRNA_0046367, circRNA_0001805, SCAR) [101]. However, hitherto, knowledge regarding the utility of lncRNAs and circRNAs encapsulated in EVs as biomarkers for MASH/MASLD is scarce.

### 4.2. Alcoholic Liver Disease

Alcoholic liver disease (ALD) is the main cause of chronic liver disease, liver fibrosis, and cirrhosis worldwide [103]. The pathophysiological mechanisms of ALD include not only the direct toxic role of alcohol but also other ethanol-induced inflammatory responses [104]. In fact, alcohol metabolism leads to the generation of oxygen free radicals, nitric oxide, and acetaldehyde, which contribute to cellular damage and liver inflammation. In this complex mechanism, RNAs, especially miRNAs, can exert an important role in regulating cytokines and other inflammatory components [104].

In both preclinical and human studies, elevated levels of circulating EVs were observed in ALD samples compared to controls [105]. This rise has also been correlated to liver damage (measured by ALT values) and is probably due to an increased activation of caspase-3 by alcohol intake [106]. The further assessment of the cargo isolated from murine serum EVs showed higher content of miR-122, miR-192, miR-30a, miR-744, miR-1246, miR-30b, miR-29a, let7f, and miR-155 [105,107,108]. These finding were partially confirmed by human trials, where EVs derived from ALD patients appeared to be enriched with miR-192, miR-30a, and miR-122, which also correlated with illness diagnosis [105]. Interestingly, increased levels of miR-122 and miR-155 were also observed in healthy individuals after binge drinking [104].

Another study performed on healthy human volunteers with binge alcohol drinking or mice after binge or chronic alcohol consumption revealed a significant rise in the number of EVs in the circulation following alcohol exposure [109]. In vitro data showed that ethanol-stimulated Huh7.5 cells presented an increased expression of Rab27b, which is critical in controlling exosome biogenesis pathways. Importantly, the EVs released showed enrichment in one the most abundant miRNAs in hepatocytes, miR-122. Thus, the EV number as well as their miR122 content are a potentially useful biomarker to monitor liver injury in ALD patients.

Analysis of EVs obtained from the plasma of patients affected by alcoholic hepatitis also revealed increased numbers of EVs that contained high levels of miR-27a as compared to those from healthy controls [110]. Mir-27a was shown to program naive monocytes to polarize into M2 macrophages, resulting in the augmented expression of several M2 macrophage surface markers, such as CD206 (mannose receptor) and CD163 (scavenger receptor), as well as the secretion of IL-10 and TGFβ and increased phagocytic activity.

Although ALD is for sure a global health problem, the difficulty in conducting clinical trials in active addicts and the lack of experimental models of advanced disease are a challenge, and little progress has been made in its cure. Thus, the analysis of the level of RNAs enclosed in EVs, such as miR-27a in combination with miR-122 as well as hepato-specific mRNAs, may develop in a “barcode” for the early detection of pathological alterations occurring specifically in ALD patients for a timely intervention. Preclinical studies are essential as they can provide indications on potential EV-RNA biomarkers for ALD. For instance, in an effort to assess the miRNA signature of EVs released from the injured hepatocytes upon intragastric infusion of ethanol upon a high-fat diet in mice, Eguchi et al. found miR-let7f, miR-29a, and miR-340 enriched in EVs released from hepatocytes into the blood under mild alcoholic steatohepatitis (ASH) compared to those of other models of chronic liver injury (bile duct ligation, non-alcoholic steatohepatitis, and obesity) [107]. Likewise, Momen-Heravi and colleagues observed a marked elevation in specific miRNAs in exosomes isolated from alcohol-fed mice and human alcoholic hepatitis patients [105]. Notably, exosomes derived from alcohol-fed mice exhibited an upregulated expression of miRNA-122, miRNA-192, and miRNA-30a. The increase in exosomal miRNA-30a and miRNA-192 observed in this study mirrored clinical data, offering further support for their potential role as biomarkers in alcoholic hepatitis [105]. These miRNAs showed high specificity to the alcohol-induced condition. Importantly, similar findings were observed in the blood of ALD patients with respect to non-alcoholics, showing the translational significance of the preclinical study. Thus, preclinical studies combined with clinical sample analysis should be encouraged, especially because it is possible to induce ALD and follow liver disease initiation and progression, from ASH to severe ALD-like alcoholic hepatitis, with the possibility of finding specific, stage-wise RNA biomarkers.

### 4.3. Autoimmune Hepatitis

Autoimmune hepatitis (AIH) is a relatively rare chronic immune–inflammatory liver disease [111]. AIH has a universal distribution, but its prevalence varies according to sex, age, and ethnicity [112]. Although it can affect individuals of various sex and age, AIH is most frequent in women with a bimodal age-related incidence curve (a first peak in children and adolescents and a second one in middle age) [112,113]. AIH is characterized by non-specific symptoms, elevated serum aminotransferases, hyperglobulinemia, presence of serum autoantibodies, and evidence of interface hepatitis [114]. Approximately 12–35% of patients are totally asymptomatic, while one-third of them already have cirrhosis at diagnosis, regardless of the presence of symptoms [112]. As an autoimmune disease, AIH involves T-cell-mediated complex immune processes, which lead to the infiltration of lymphocytes, macrophages, and plasma cells into the liver [115]. The different cell types involved in AIH can communicate with each other through exosomes or EVs in a bidirectional way, that is, injured hepatocytes can release EVs with cargos affecting the recruitment and conditioning of immune cells and vice versa [116]. Thus, quantifying and profiling these EVs can lead to the discovery of an EV-based RNA signature specific to AIH and, hence, aid in its early diagnosis. However, studies on this matter are still too few and often analyzed small cohorts of patients because of the rarity of the disease.

Paluschinski et al. recently observed that circulating EVs were significantly more numerous and smaller in AIH patients compared to healthy subjects, although these characteristics did not correlate with liver injury [117]. Regarding EV content, available data are mainly focused on miRNAs. In fact, recent studies have shown that subjects with AIH presented an altered expression of several EV-enclosed miRNAs with respect to healthy donors. In particular, miR-142-3p, miR-10a, and miR-223 were significantly upregulated. On the other hand, miR-150, miR-15a, and miR-21 were significantly enriched in AIH patients’ serum-derived EVs compared to those of non-AIH subjects (AIH vs. NAFLD, AIH vs. healthy controls, and NAFLD vs. healthy controls) [117]. In another study, Abe et al. showed that 3 miRNAs encapsulated in circulating EVs (miR-7855-5p, miR-6806-5p, and miR-557) out of 2569 obtained with miRNA array profiling and could discriminate AIH patients from healthy controls [118]. In particular, EV-enclosed miR-557 showed significantly high specificity as a diagnostic or relapse marker in AIH versus NASH, Primary Biliary Cirrhosis, or healthy controls. An EV level of miR-557 > 7.69 copies/μL was associated with a higher risk of relapse, indicating that serum EV RNA levels may also help in predicting disease relapses [118].

While data regarding other circulating RNAs, such as miR-155 and miR-122, remain to be confirmed in the setting of AIH, promising findings came from miR-223, which plays an essential role in liver homeostasis and chronic liver diseases such as NAFLD during which miR-223-rich EVs are released from neutrophils [119,120]. In fact, the use of miR-223-containing exosomes seemed to protect against liver injury through a reduction in pro-inflammatory cytokines in a model of experimentally induced AIH (S100-induced mice model), hence warranting further studies on the biomarker potential of this miRNA vehicle by circulating EVs [121,122]. In conclusion, the participation of EV-RNAs in the induction and pathogenesis of AIH remains currently understudied and requires further investigations.

### 4.4. Chronic Viral Hepatitis

Viral hepatitis, caused by infection with carcinogenic viruses such as HBV or HCV, causes chronic inflammation and induces fibrotic changes in the liver and is a major risk factor for HCC [123]. Viral infections can be diagnosed with serological assays such as those measuring the HB-core antibody, HB-surface antibody, and HB-surface antigen in serum samples to detect subjects exposed to HBV or anti-HCV antibodies in HCV-exposed patients [124]. Only a fraction of individuals infected with HCV may progress to severe hepatic cirrhosis or HCC, and predicting the outcome is challenging. There is thus the necessity to find biomarkers capable of predicting the risk of viral hepatitis progression. EV-enclosed ncRNAs offer such an opportunity. Numerous studies have shed light on the significant role of miRNAs within EVs in the progression of viral hepatitis. For instance, HCV-RNA has been linked with miR-122 within HCV-infected hepatocytes, and levels of miR-19a in serum EVs of chronic HCV patients with fibrosis were notably increased compared to those of healthy subjects [125]. In a study on 39 early-stage fibrotic patients (F0-F2) with chronic HBV or HCV infection and 14 control subjects who underwent transient elastography (Fibroscan), Lambrecht et al. showed that in circulating EVs, miRNA-192, miR-200b, miR-92a, and miR-150 were significantly downregulated in both HBV and HCV patients, while total plasma samples were significantly enriched in miRNA-200b and miRNA-122 under both conditions [126]. Thus, the type of liquid biopsy considered in the clinic is very important, with EVs providing non-invasive biomarker panels, which could develop into bioassays for detecting early fibrosis and small fluctuations in the fibrotic liver. An increasingly recognized role of EVs in viral hepatitis is underscored by the finding that lncRNAs contained within EVs also significantly contribute to predicting the evolution of chronic viral hepatitis to cirrhosis. For example, *lncRNA-HEIH* was found to be enriched in the EVs of HCV-related HCC patients versus control subjects [127]. This allowed the differential diagnosis of HCV-related HCC with respect to chronic hepatitis B and liver cirrhosis [128]. Further studies are needed to fully characterize the ncRNA contents of EVs in order to find a panel of biomarkers able to discriminate among patients infected with HCV or HBV and showing evolution into HCC versus those with a clinically stable situation.

### 4.5. Cholestatic Liver Diseases

Cholestatic liver diseases are an important clinical problem, representing 10% of all liver disease and impinging heavily on the health care system [129]. The obstruction of bile flow and the accumulation of bile acids, which are characteristic features of chronic cholestasis-induced liver diseases, can cause cellular damage which can progressively lead to inflammation and fibrosis [130]. Following injury, cholangiocytes, hepatic stellate cells, and portal fibroblasts, which are the main cell types involved in cholestasis-related pathogenesis, undergo activation and proliferation [131]. Fibrosis slowly evolves, if left unchecked, to liver failure and cancer [132]. The impaired bile flow may be caused by the obstruction of bile ducts by gallstones, local tumor impingement, and drug-induced toxicity, while genetic defects are responsible for cholestatic diseases such as primary biliary cholangitis (PBC), biliary atresia, and primary sclerosing cholangitis (PSC) [133]. Liver transplantation is the only definitive solution for end-stage patients [134]. Monitoring cholestasis-induced early fibrotic changes is not easy; liver biopsy is the gold standard, but it has poor patient compliance and a high risk of complications. Furthermore, as liver biopsy does not always allow for the early detection of fibrosis, new non-invasive alternatives are necessary [135].

EV-enclosed RNAs are promising also for the diagnosis of chronic cholestatic diseases, as well as for their evolution from fibrosis to cholangiocarcinoma (CCA). At the preclinical level, in an effort to search for EV contents capable of detecting early fibrosis, for which limited diagnostic tools are available, we employed several mouse models of cholestasis-induced liver fibrosis. Serum EV analysis with RNA sequencing (small RNA-seq and whole transcriptome) revealed that miR-192-5p, miR-194-5p, miR-22-3p, and miR-29a-3p and *Hp* mRNA were significantly enriched in the circulating EVs of cholestatic mice with respect to non-cholestatic ones. Importantly, the level of these EV-RNA species correlated with the development of cholestasis-induced liver fibrosis [53]. Different RNA species were identified in circulating EVs with whole-transcriptome analysis, in particular the terms “proteins”, “lincRNA”, “snoRNA”, “snRNA”, “antisense”, “miRNA”, “scaRNA”, “misc_rna”, “sense_intronic”, and “sense_overlapping”, which require further investigation as potential RNA biomarkers for cholestasis-induced liver fibrosis [53].

H19 lncRNA has been shown to be present in EVs released by cholangiocytes and to exacerbate cholestatic injury. In Mdr2-/-mice, H19 lncRNA in serum exosomes gradually increased with disease progression, indicating that this biomolecule can develop into an RNA biomarker for cholestatic injury and its progression into more severe disease. Moreover, in biliary cholangitis and biliary atresia patients, the serum level of EV-carried H19 was found to correlate with the severity of fibrotic liver injury [136].

In an effort to find RNA biomarkers for CCA development, Lapitz et al. analyzed the RNA profile of serum and urine EVs of ulcerative colitis, primary sclerosing cholangitis, and CCA patients versus healthy individuals [137]. Regarding serum, comparison of CCA patients’ and healthy individuals’ EV-derived RNA revealed the RNA candidate mRNAs ring finger and FYVE-like domain containing E3 ubiquitin protein ligase (RFFL), olfactory receptor family 4 subfamily F member 3 (OR4F3), and the family with sequence similarity 107 member B (FAM107B) as well as ncRNAs PMS1 homolog 2 mismatch repair system component pseudogene 4 (PMS2L4), miR-604, and SNORA58. Moreover, several RNAs were also differentially present in EVs derived from CCA versus PSC, in particular, the mRNA transcripts paraoxonase 1 (PON1), activating transcription factor 4 (ATF4), and phosphoglycerate dehydrogenase (PHGDH), the lncRNAs metastasis-associated lung adenocarcinoma transcript 1 (MALAT1) and LOC100190986, and the small nucleolar RNA (snoRNA) SNORA11B. These serum EV-derived differentially expressed RNA molecules also presented promising biomarker candidates for the diagnosis of PSC with respect to ulcerative colitis and healthy subjects. Several RNA species were differentially present in urine-derived EVs in CCA patients compared to healthy individuals, such as the mRNAs MAP6 domain containing 1 (MAP6D1) and Ras-related GTP-binding D (RRAGD) and the long non-coding RNAs (lncRNAs) HLA complex group 4 (HCG4) and the lncRNA LOC100134868. Promising RNA biomarker candidates differentiating between PSC and CCA were also detected from urine-derived EVs [137]. On the whole, albeit still few, these studies indicate promising RNA biomarker candidates that deserve further investigation in the clinical setting.

### 4.6. Liver Fibrosis

The incessant inflammation in the hepatic tissue and inflammatory cytokine production by damaged hepatic cells, accompanied by the destruction and regeneration of the liver parenchyma, leads to enhanced deposition of the extracellular matrix or fibrosis [1]. Diagnosing liver fibrosis very early, when it is still reversible, is essential to prevent deterioration into cirrhosis. The gold standard for detecting liver fibrosis is the histological analysis of biopsy specimens; however, the invasive nature of the procedure and the associated risks reduce patient compliance [138]. The utility of circulating biomarkers including circulating proteins, nucleic acids, and metabolites, as well as RNA for predicting liver fibrosis, has been described in several studies, including [139,140,141,142]. EV-enclosed ncRNAs have also been investigated as potential biomarkers for the grading of liver fibrosis [143]. In a preclinical model of carbon tetrachloride (CCl_4_)-induced liver fibrosis, it was found that the increase in EV miR-155 correlated with the degree of hepatic necrosis and liver fibrosis [144]. The diagnostic accuracy increased when combined with circulating protein (for example, AST) levels. Indications also come from preclinical rodent models. In mice subjected to CCl_4_ treatment, the levels of miR-214 together with Twist1 and connective tissue growth factor (CCN2) increased in circulating exosomes versus control mice and correlated with fibrosis severity, indicating the potentiality of this panel of biomolecules for fibrosis diagnosis and grading [63]. Moreover, miR-34c, miR-151-3p, miR-483-5p, and miR-532-5p were significantly lower in the EVs of fibrotic mice (CCl_4_-induced) and hepatic fibrosis patients compared to in those of their respective controls [145]. In another study, Lee et al. found that the treatment of hepatocytes with lipotoxic palmitic acid induced the levels of EVs and enclosed miR-122 and miR-192. These miRNAs are involved in the progression of steatohepatitis to liver fibrosis by regulating the expression of several fibrosis-related genes including α smooth muscle actin (αSMA) and collagen type 1 alpha 1 (col1α1) in the hepatic stellate cell line LX-2 [146].

Regarding lncRNAs, in subjects with biliary cholangitis and biliary atresia, there is a reported correlation between the severity of fibrotic liver injury and the level of EV-carried lncRNA H19 in the serum [136]. The DNA methyltransferase 1 (DNMT1)-mediated epigenetic silencing of lncRNA H19 is associated with the activation of hepatic stellate cells, which is involved in liver fibrosis [147]. In a mouse model of liver fibrosis induced by arsenite, Dai et al. found an increase in the level of lncRNA-MALAT1 in the EVs of treated mice, with respect to those of controls [148]. Arsenite causes oxidative stress and hepatic stellate cell activation, thereby inducing liver fibrosis. Interestingly, EV lncRNA-MALAT1 levels were higher in arsenic-exposed individuals with respect to healthy volunteers. With high-throughput technologies, the number of EV-miRNAs and EV-lncRNAs identified as possible biomarkers for liver fibrosis staging is bound to increase.

### 4.7. HCC

The potential of lncRNAs found in circulating EVs as non-invasive biomarkers has also been evidenced, mainly in the diagnosis of HCC and cholangiocarcinoma (described above for the latter), and extensively described in a recent review by Yang et al. [149,150]. In the study by Kim et al., the authors showed that the HCC-driver oncogenic lncRNA candidates, EV-MALAT1, EV-DLEU2, EV-HOTTIP, and EV-SNHG1, were detected in serum EVs and were promising as a panel of biomarkers in the diagnosis of HCC and showed superior sensitivity compared to Alpha-fetoprotein (AFP) [151]. Importantly, two of these lncRNAs, EV-MALAT1 and EV-SNHG1, were also able to detect the early stages of HCC. EV-lncRNAs coupled with EV-miRNAs analysis as a panel of biomarkers can also offer improvements in the diagnosis of HCC. For example, Matboli et al. extracted lncRNAs involved in HCC followed by the search of selected lncRNAs in EVs isolated from the serum of HCC patients compared to non-HCC ones. This study revealed that the presence of ncRNAs hsa-miRNA-1298 and lncRNA-RP11-583F2.2 in EVs had better sensitivity and specificity in detecting HCC with respect to AFP [152].

Lee et al. investigated the role of exosomal ncRNAs in the prognosis of HCC [153]. In particular, they analyzed the level of EV-enclosed miRNA-21 and lncRNA-AT in the serum of HCC patients versus healthy individuals. It was found that these two ncRNAs were significantly enriched in the EVs of HCC patients with higher mortality and lower disease-free survival with respect to those with lower levels of these two EV-RNA species, highlighting the potential of not only miRNAs but also lncRNAs as prognostic RNA biomarkers for HCC outcome.

Importantly, classification on the basis of cancer-related RNA modification patterns may also be of prognostic value for HCC [154]. RNA backbone modifications linked to various liver conditions, including hepatitis B and C, MASLD, and HCC, have been unveiled by recent technological advancements [155,156]. N6-methyladenosine (m6A) has emerged as a pivotal post-transcriptional modification, notably fostering MASLD development through adipogenesis, lipid metabolism, inflammation, and insulin resistance [157]. Moreover, m6A modification plays a crucial role in hepatitis B virus (HBV) replication and immunity, thereby contributing to HBV-induced HCC pathogenesis [158]. Similarly, analogous mechanisms may be implicated in hepatitis C virus (HCV) infection and m6A-mediated immune system suppression, potentially leading to chronic hepatitis and HCC [159]. Dysregulated m6A RNA and enzyme profiles observed in HCC suggest a close correlation with disease progression due to altered messenger RNAs (mRNAs) and ncRNAs [156]. A recent study by Zongqiang et al. investigated the role of plasma-derived exosome-encapsulated m6A-circCCAR1, which promotes CD8+ T-cell dysfunction and anti-PD1 resistance in HCC [160]. These findings underscore the potential of targeting post-transcriptional modifications for future CLD diagnoses and therapeutic interventions (Table 3).

## 5. Challenges and Future Perspectives

Liquid biopsy has emerged as an efficient non-invasive approach for the early detection of CLD, with circulating EVs being accepted as an optimal reservoir of biomarkers. The EV protein and metabolite content are promising, but they do not always address the need to find specific CLD biomarkers (diseased tissue-derived) that distinguish diseases according to etiology. Combining these biomolecules with EV-derived RNAs can help in devising a panel of biomarkers for the diagnosis or prognosis of CLD according to etiology. Moreover, as soon as liver injury occurs, EVs are rapidly released into the circulation, harboring RNA and metabolites that are present in the cytoplasm. While translation is a rapid process, it generally occurs at a slightly slower rate compared to transcription. Thus, the RNA molecules present in the EVs at very early time points after injury are more likely to reflect the very early pathological situation of the liver, with respect to the metabolites.

To date, mRNAs and miRNAs, together with lncRNAs, remain the most studied EV-carried RNA biomarkers of chronic liver diseases. The advancement in high-throughput sequencing has put different types of EV-enclosed ncRNAs in the limelight for biomarker research. Of these, piRNAs have recently attracted considerable interest. PiRNAs enclosed in EVs could develop into useful diagnostic biomarkers to distinguish, for instance, HCC patients from non-tumor ones. In this context, Rui et al. compared, using small RNA sequencing, serum EVs (exosomes) derived from 125 HCC patients and 44 non-tumor controls [161]. Five differentially expressed piRNAs (piR-15254, piR-1029, piR-35395, novel-piR-32132, and novel-piR-43597) were present at significantly higher levels in the HCC samples with respect to the controls, which when combined presented a superior AUROC (area under the receiver operating characteristic) with respect to the single piRNAs, revealing the potential diagnostic value of this piRNA signature in the detection of HCC, including that with low tumor burden. Differentially expressed piRNAs (piR-2660989, piR-10506469, piR-20548188, piR-10822895, piR-hsa-23209, and piR-18044111) were also found in EVs isolated from blood samples of CCA and gallbladder carcinoma (GBC) patients versus healthy controls [162]. Interestingly, of these upregulated piRNAs, two piRNAs (piR-10506469 and piR-20548188) significantly decreased in the circulating EVs of the CCA and GBC patients 1 week after surgeries, indicating that these piRNAs are promising candidate biomarkers.

Circular RNA (circRNA) and transfer RNA-derived small RNA (tsRNA) present in circulating EVs also have promising diagnostic potential in the setting of HCC. For instance, Sun et al. found that the combined analysis of three circRNAs (hsa_circ_0004001, hsa_circ_0004123, and hsa_circ_0075792) significantly improved the diagnosis of HCC [163]. Regarding tsRNA, Zhu et al. showed that several tsRNAs, in particular, tRNA-ValTAC-3, tRNA-GlyTCC-5, tRNA-ValAAC-5, and tRNA-GluCTC-5, were differentially enriched in the plasma-derived EVs of HCC patients with respect to healthy donors, pointing to the fact that tsRNAs could develop into new diagnostic biomarkers for HCC [164].

Microfluidic devices coupled with organ-on-chip systems are expected to boost the biomarker search for not only CLD but also for other diseases [165]. Challenges regarding the cost-effectiveness of the studies, requirement for trained personnel, sampling, and conservation need to be tackled before EV-borne RNA biomarkers can be routinely applied in the clinic.

The exploration of EV-encapsulated ncRNA as a biomarker, as previously discussed, is still in its nascent phases and necessitates further refinement. Research suggests that dosing multiple EV ncRNAs with conventional serum markers could markedly enhance diagnostic, prognostic, or predictive precision. Several points need to be addressed before EV-enclosed RNAs can be introduced as biomarkers into the clinical routine, including the need to find universally acknowledged reference RNAs, as well as the necessity to establish standardized protocols across research institutions, especially regarding the internal reference RNAs [150]. Moreover, most studies have been conducted on animal models or limited patient groups, emphasizing the need to validate the association between specific EV-derived ncRNAs and CLD in broader patient cohorts in the future [150].

## 6. Conclusions

Despite the extensive presence of different species of RNAs circulating in the blood, the potentiality of EV-enclosed RNAs as biomarkers cannot be replaced by cell-free RNAs due to the enhanced stability of the RNAs encapsulated inside the lipid bilayers of these nanoparticles. While there is still much work to be conducted to implement the application of EVs as biomarkers for CLD in clinical settings, research in this field should be strongly encouraged. Efforts should be focused on directing research towards identifying biomarkers for the detection of patients at the highest risk of disease progression. Additionally, there should be an emphasis on serial sampling and longitudinal monitoring of liver diseases to prevent their advancement into chronic or life-threatening conditions.

## Figures and Tables

**Table 1:** Examples of biomolecules experimentally identified in liver-derived EVs.

Liver Cell Type *	Examples of Biomolecules Enriched in Liver-Derived EVs	References
Hepatocytes	Asialoglycoprotein receptor (ASGPR); cytochrome P450 isoforms; secreted proteins such as those of the complement system and coagulation pathway, as well as apolipoproteins; drug-metabolizing enzymes, including uridine diphosphate glucuronosyltransferases (UGTs), alcohol dehydrogenase-1 (ADH1), and glutathione S-transferase (GST); albumin (ALB); haptoglobin (HP), sphingosine-kinase 2 (SK2); miRNAs such as miRNA-122, -192, and -128-3p	[50,51,52,53,54,55,56,57]
Cholangiocytes	polycystin-1; growth factors such as FGF7; EGFR ligands; long ncRNA H19; Hedgehog ligands	[58,59,60,61]
Hepatic stellate cells	PDGFRα; Hedgehog ligand; Twist-1; connective tissue growth factor; miR-214; miR17-92 cluster	[61,62,63,64,65,66]
LSECs	SK1; fatty acid-binding protein 4	[67]

* To date, no characterization of Kupffer-derived EVs have been reported, but these cells may release EVs with characteristics similar to those of other types of macrophages [68].

**Table 2:** Types of EV-enclosed biomolecules and their characterization methods.

Biomolecules	Characterization Methods
RNA	Sequencing (small RNA-seq; whole-transcriptome RNA-seq); quantitative real-time PCR
Protein	Western blotting; ELISA; proteomics; flow cytometry; protein quantification
Lipids	Lipidomics
Metabolites	Nuclear Magnetic Resonance; Mass Spectrometry

**Table 3:** Examples of dysregulated non-coding RNA biomarkers in CLD.

Liver Disease	Biomarker	Modification (Role)
MASLD	EVs miRNAs lncRNA circRNA	↑ total amount * ↑ miR-122, miR-192, miR-128-3p (disease severity) ↓ miR-135a-3p, miR-129b-5p, miR-504-3p * ↑ NEAT1, MEG3, MALAT1 ↑ SCAR, circRNA_0046367, circRNA_001805
ALD	EVs miRNAs	↑ total amount ↑ miR-122, miR-192, miR-30a, miR-27a ↑ miR-744, miR-1264, miR-30b, miR-29a, miR-155 *
AIH	EVs miRNAs	↑ total amount and ↓ size ↑ miR-142-3p, miR-10a, miR-223, miR-150, miR-15a, miR-21 ↑ miR-557, miR-7855, miR-6806-5p (disease diagnosis) ↑ miR-557 (disease relapses) ↑ miR-223 (protective vs. disease) *
Viral hepatitis HCV HCV and HBV	miRNAs lncRNA	↑ miR-19a ↓ miRNA-192, miR-200b, miR-92a and miR-150 ↑ *lncRNA-HEIH*
PSC/PBC	miRNAs lncRNAs mRNAs	↑ miR-192-5p, miR-194-5p, miR-22-3p, miR-29a-3p * ↑ H19 (disease severity) * ↑ MALT1, LOC100190986 (disease diagnosis) ↑ PON1, ATF4, PHGDH (disease diagnosis)
Fibrosis	mRNAs lncRNA	↑ miR-122, ↑ miR-192, ↑ miR-155, ↑ miR-214, ↓ miR-34c, ↓ miR-151-3p, ↓ miR-483-5p, ↓ miR-532-5p ↓ H19, ↑ MALAT-1
HCC	miRNAs lncRNAs	↑ miR-223, let-7e-5p, miR-486-3p (AR risk) ↓ miR-199a-3p, miR-152-3p (AR risk) ↑ miR-301a (death for AR) ↑ miR-718 (HCC recurrence) ↑ lncRNA FAL1 ↑ RP11-85G21.1 (lnc85)

* Only preclinical trials available. MASLD: metabolic dysfunction-associated steatotic liver disease; ALD: alcoholic liver disease; AIH: autoimmune hepatitis; PSC: primary sclerosing cholangitis; PBC: primary biliary cholangitis; LT: liver transplantation; AR: acute rejection; HCC: hepatocellular carcinoma (↑: increased expression; ↓ decreased expression).

## Supplementary Materials

The following supporting information can be downloaded at: https://www.mdpi.com/article/10.3390/biom14030277/s1, Table S1: Examples of EV biodistribution time in the liver in mouse models. References [70,166,167,168] are cited in Supplementary Materials.
