# Supplementary material for: Extracellular Vesicles as Delivery Vehicles for Non-Coding RNAs: Potential Biomarkers for Chronic Liver Diseases
Source: Biomolecules. 2024 Feb 26;14(3):277. doi: 10.3390/biom14030277 (PMC10967855; doi:10.3390/biom14030277)
Supplement: Supplementary file 1 [file biomolecules-14-00277-s001.zip › biomolecules-2809939-supplementary.pdf]

Table S1. Examples of EV biodistribution time in the liver in mouse models.

EV source	EV concentration/ type of imaging	EV administration route	Type of Liver disease/cell type uptaking EVs	Time taken to reach the liver/Permanence in the liver/ in vivo or ex vivo analysis	References
Red blood cell<br>-EVs loaded with miR-negative control (NC)-ASOs or miR-155-ASOs	100 μg ≈ 2.3 × 1010 particles/ IVIS imaging	Intravenous	Acute liver failure/macrophages	12 hours/24 hours (last time point analysed)/ex vivo	[166]
MLP29 (murine liver-derived cells)- [124 I] NaI-labelled EVs	1.8 ± 0.5 MBq/ PET-CT imaging<br><br>0.6 ± 0.2 MBq/ automatic gamma counter	Intravenous<br><br>Into the hock	Healthy/ n.a.	15 mins/72 hours (last time point analysed)/in vivo	[167]
HEK293T- 99mTc-HYNIC-Duramycin labeled EVs	12 ± 3 MBq/ SPECT/CT imaging	Intravenous	chronic hyperlipidemia/ Kupffer cells and liver sinusoidal endothelial cells (from in vitro data)	60mins/90 mins (last time point analysed)/ in vivo	[70]
Thyroid cancer (CAL-62) and breast cancer (MDA-MB-231) cells<br>-Rluc- labelled CAL-62<br>EVs or Rluc- labelled MDA-231 EVs	25 μg of protein/ IVIS Lumina II	Intravenous	Naïve nude mice	10 mins, with a peak at 30 mins /9 days/ in vivo	[168]

PET: Positron Emitted Tomography; CT: Computed Tomography, MBq: Megabecquerel; n.a.: not available.
